# Convergence and parallelism in the evolution of plant metabolism

**DOI:** 10.1111/jipb.70236

**Published:** 2026-03-19

**Authors:** Federico Scossa, Mustafa Bulut, Thomas Naake, John C. D'Auria, Alisdair R. Fernie

**Affiliations:** ^1^ Max‐Planck Institut für Molekulare Pflanzenphysiologie Potsdam‐Golm D‐14476 Germany; ^2^ Leibniz Institute of Plant Biochemistry Halle (Saale) 06120 Germany; ^3^ Physikalisch‐Technische Bundesanstalt (PTB) Braunschweig 38116 Germany; ^4^ Department of Biochemistry and Bioinformatics Technische Universität Braunschweig Braunschweig 38106 Germany; ^5^ Leibniz Institute of Crop Plant Genetics and Crop Plant Research (IPK) OT Gatersleben Seeland 06466 Germany

**Keywords:** adaptation, convergent evolution, domestication, metabolism, natural selection, parallelism, plants

## Abstract

Convergence and parallelism are contentious terms in evolutionary biology, but both denote essentially a ubiquitous phenomenon: The occurrence of similar phenotypes, in different evolutionary lineages, in a way that cannot be easily reconducted to descent from a shared ancestor. In this article, we trace the historical definitions of the two terms and the current conceptual frameworks to classify instances of repeated evolution, presenting the limits of these approaches in considering convergence and parallelism as a strict dichotomy rather than as part of a continuum along the spectrum of phenotypic similarity. We then present cases of convergence—broadly defined—from plant domestication and specialized metabolism, with the objective of understanding the intricacies between natural selection, constraints and drift underlying the recurrent appearance of complex traits.

## Graphical abstract

Similar traits in different organisms may originate from shared ancestry or evolve independently. The terminology used to define phenotypic similarity is often confusing. This review attempts to clarify the definitions and present examples from plant domestication and specialized metabolism to explain how complex traits evolve repeatedly in plants.

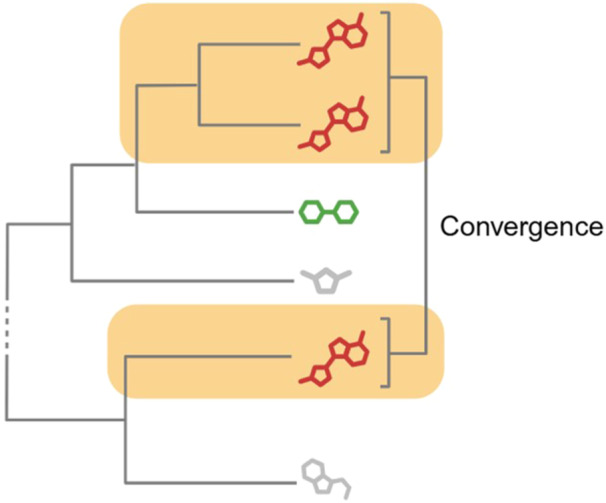

## A MATTER OF DEFINITIONS: ARE CONVERGENCE AND PARALLELISM REALLY TWO DIFFERENT PHENOMENA?

In evolutionary biology, the debate on the semantic distinction between the terms “convergence” and “parallelism” has been the subject of decades of discussions (Arendt and Reznick, 2008; Leander, 2008; Scotland, 2011; Pearce, 2012; Bolnick et al., 2018; Cerca, 2023; James et al., 2023). In this first section of the review, we trace the history of usage of the two terms and, in doing so, we will attempt to clarify what we believe is the most appropriate terminology. We will also attempt to address the focal question that we used as a heading for this section, that is, whether “convergence” and “parallelism” can be really distinguished by some well‐defined mechanisms or characteristics, or are, at best, synonyms to denote essentially the same phenomenon along the spectrum of the various instances of phenotypic similarity. Although our focus in the later sections of this review is on plant metabolism, our general discussion below of the terminology is of general value and can be applied to any phenotype.

**Figure 1:**
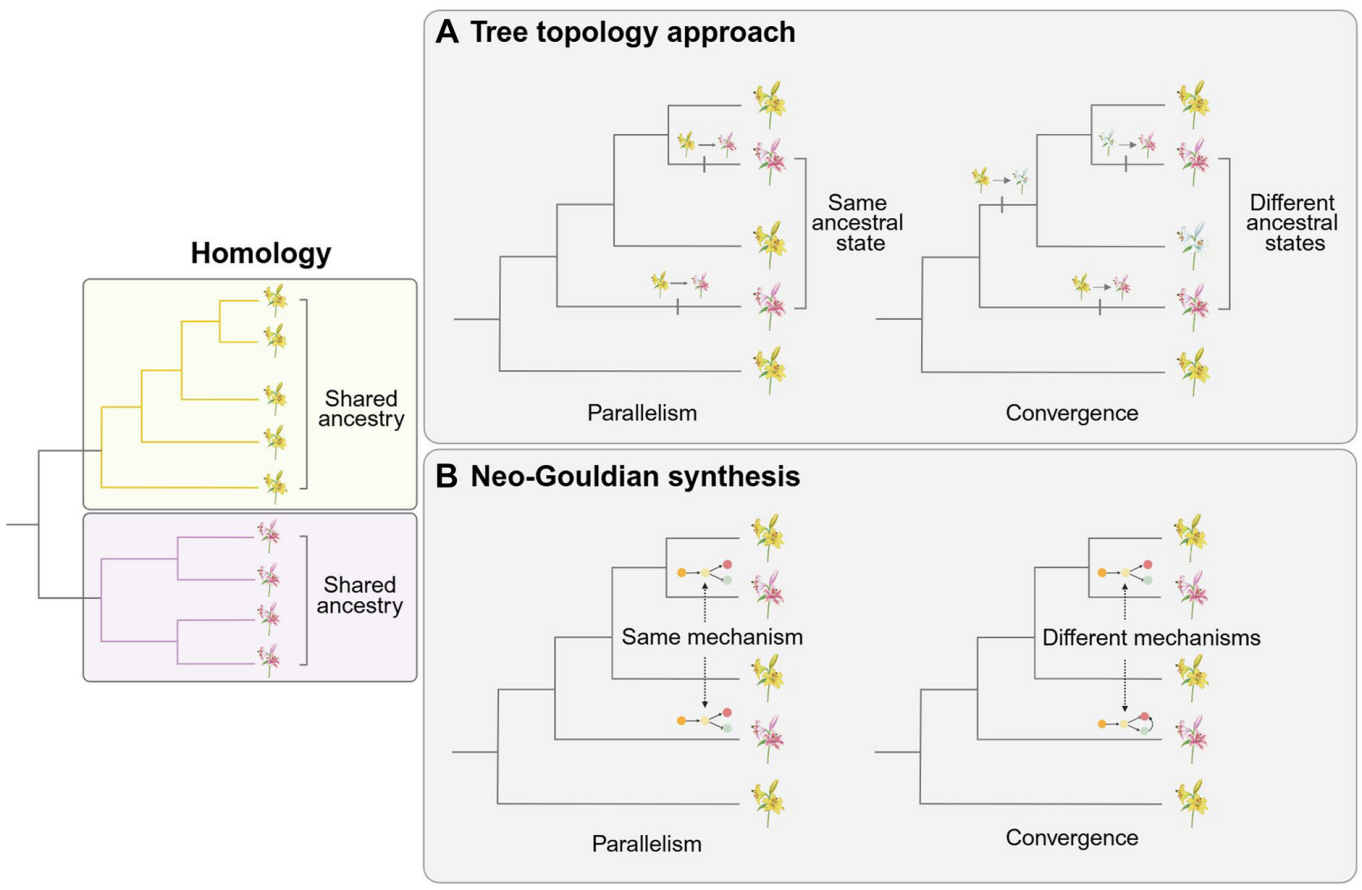
Conceptual frameworks for defining parallelism and convergence **(A**, **B)** Similar phenotypes may arise from shared ancestry (homologous traits) or emerge through homoplasy (similarity not derived from shared ancestry). Homoplastic traits can be reconducted to cases of parallel or convergent evolution. In the tree topology approach **(A)** (Pearce, 2012), parallelism is observed whether the similar phenotypes (the pink flowers) arise from the same ancestral state (here, the yellow flower). Convergence, on the other hand, occurs whether the pink flowers evolve from different ancestral states. The Neo‐Gouldian synthesis **(B)** (Gould, 2002) distinguishes instead whether the mechanisms generating the similar phenotypes are homologous (parallelism) or non‐homologous (convergence). A simple biochemical pathway is represented as an example of mechanism: in parallelism, the pathway follows the same steps and is governed by homologous genes, while in convergence, the two pathways leading to the pink flower color are different (non‐homologous genes and different intermediates). Figure created using BioRender.

The terms “convergence” and “parallelism” were initially used starting from the late 1800s in papers describing the presence of similar structures in comparative anatomy of mammals (Scott, 1891; Osborn, 1902). The intended use was to distinguish morphological similarity observed in closely (parallelism) or in distantly related groups (convergence). Since then, this original distinction was considered a contentious issue in the evolutionary biology community and subjected to revisions and adjustments, with some authors even proposing to abandon this distinction in favor of adopting more generic terms (either only “convergence” (Arendt and Reznick, 2008) or “replicated evolution” (James et al., 2023) to denote all cases of phenotypic similarity). The dispute over the meaning of these two terms reflected the debate (which is still largely present today; see Laland et al. (2014)) between the “externalists”, those who consider natural selection as the only force determining homoplasy (Table 1), versus those, predominantly from the developmental biology field, who instead also invoke the contribution of internal factors (constraints) to the emergence of similarity (Wake et al., 2011). The issue might seem purely speculative at first, but it relates to one of the oldest problems of evolutionary theory: the role, if any, of internal constraints in the emergence of phenotypic variation (Gould, 1989). Whether similar traits, which are not the result of shared ancestry, are observed in different lineages, externalists consider natural selection to be the only driving force (convergence), while other authors consider the similarity to be the product of both internal and external forces (parallelism) (Wake, 1991; Losos, 2011; Wake et al., 2011; Pearce, 2012). Apart from these views, David Jablonski proposed a simple clear‐cut criterion to distinguish parallelism from convergence: his approach is based solely on tree topology and is completely agnostic to the potential similarity of developmental mechanisms (Pearce, 2021; Figure 1). An alternative to the topological approach was proposed by Stephen Jay Gould with the inclusion of the concept of homologous “underlying generators” (i.e., the internal developmental mechanisms) to distinguish parallelism (homologous mechanisms) from convergence (non‐homologous mechanisms) (Gould, 2002; Figure 1).

**Table 1:** Glossary of some ambiguous terms in parallel and convergent evolution

Term	Definition
Homologous traits	Characters in different organisms that are similar because they were inherited from a common ancestor that also had that character (shared ancestry).
Homoplasy	Similarity in a trait that cannot be explained by descent from a common ancestor. Historically, homoplastic traits (as opposed to homologous) have been classified as convergent or parallel whether they occur in related (convergent) or in unrelated groups (parallel) (Haldane, 1932). This classification coincides with the oldest reported use of the two terms (Osborn, 1902), and is based essentially on strict taxonomic criteria, agnostic to the potential similarity in developmental mechanisms generating the traits but also to ancestral character states. This historical early definition has been the subject of decades of intense debate and subsequent revisions proposed by various authors (see text for details).
Independent evolution	The emergence of a trait in two or more lineages by means that cannot be explained by shared ancestry. Used as a synonym of replicated evolution *sensu* (James et al., 2023) and as an umbrella term including both parallel and convergent evolution.

However, all of the above criteria have been the subject of criticisms. Distinguishing convergence from parallelism simply on the basis of where homoplasy (Table 1) occurs (in non‐better defined “related” vs. “unrelated organisms”) is a weak criterion. It does not specify at which phylogenetic distance two organisms become “unrelated”, nor does it define, for example, whether two organisms in the same genus are considered “related”. The topological approach, while providing a clear‐cut distinction with the inclusion of the ancestral character states, totally ignores similarities in developmental and genetic mechanisms underlying homoplasy. The Neo‐Gouldian synthesis, on the other hand, while also considering developmental homology, is generic in defining the hierarchical level at which two mechanisms can be defined as homologous: exactly the same mutation? In the same gene? Or homology on a pathway level?

Many other recent compelling arguments have been put forward that suggest that the distinction between “convergence” and “parallelism” does not make a lot of sense. Despite the historical definitions and the approaches outlined above, a large part of the biology community simply adopts “convergence” whether similar phenotypes arise in distantly related organisms and “parallelism” whether they occur in closely related taxa. However, many examples in the literature are confirming that even in different populations of the same species, different genetic mechanisms explain the same phenotype (see Wozniak et al. (2024), discussed further below, and Hoekstra et al. (2006), Ramos et al. (2025)). On the other hand, genomic and gene expression similarities in closely related species, or within populations of the same species, may point to the same genes and mechanisms underlying homoplasy, thus reinforcing the idea that parallelism is sustained by the same genetic mechanisms. Examples of this include the flower color transitions in *Ipomoea* (Des Marais and Rausher, 2010), *Iochroma* (Smith and Rausher, 2011), and *Mimulus* (Wenzell et al., 2025), the evolution of storage roots in Convolvulaceae (Eserman et al., 2018), and many other studies in the genetics of adaptation to various environmental conditions (Van Etten et al., 2020; Bohutinska et al., 2021; Konecna et al., 2021; Wang et al., 2021; Wos et al., 2021). Moreover, many examples exist wherein the same causal mutation has been fixed in distant species. For example, the same amino acid substitution was found to be responsible for pigmentation in beach mice and mammoths (Hoekstra et al., 2006; Rompler et al., 2006). Similarly, in plants, there are a wide range of such instances, including the aromatic amino acid decarboxylase proteins (Torrens‐Spence et al., 2020), cytochrome P450s that catalyze oxidative 5,6‐spiroketalization of cholesterol (Christ et al., 2019), rosmarinic acid biosynthesis (Levsh et al., 2019), caffeine biosynthesis (Huang et al., 2016; O'Donnell et al., 2021), *Hyoscyamus muticus* premnaspirodiene synthase (HPS), and *Nicotiana tabacum* 5‐epiaristolochene synthase (TEAS), which produce a mixture of diverse major and minor products that provide defense in Solanaceae (Weng et al., 2012). Moreover, considerable evidence has accumulated of convergent evolution in flavonoid and lignan biosynthesis (Weng et al., 2011; Weng and Noel, 2013), as well as the parallel evolution of the enzymes catalyzing the synthesis of chloroalkaloid (−)‐acutumine in both plants and bacteria (Kim et al., 2020). In the case of primary metabolism, the canonical cases center around the mode of photosynthesis, with the C4 trait having evolved independently at least 61 times (Sage, 2017) and Crassulacean Acid Metabolism (CAM) having independently evolved at least 66 times (Gilman et al., 2023).

Returning to our discussion on terminology, further problems in adopting a clear‐cut distinction of the two terms are in how close species need to be to call it “parallelism” and at which phylogenetic distance is it more likely that different genetic mechanisms are operating to achieve the same phenotype? Placing this question in simpler terms, how deep are we allowed to go in the evolutionary tree to consider two organisms as related? This problem of “where to draw the line” is also recalled in (Cerca, 2023), in which a valiant attempt is made to clarify the terminology but some gray areas are also introduced in the classification of parallel and convergent evolution, given that the proposed distinction is based on the *phenotypic ancestral states* (which cannot be inferred for all phenotypes) and on the concept of *geometric phenotypic trajectories*, which do not necessarily describe evolutionary dynamics (Bolnick et al., 2018).

The problem of “where to draw the line” is also present in another largely used definition of convergence and parallelism. As we mentioned above, the Neo‐Gouldian synthesis (Pearce, 2012) as well as other authors (Arendt and Reznick, 2008) use the term “parallelism” if the same genetic/developmental mechanisms underlie homoplasy, and “convergence” if, instead, phenotypic similarity is generated through different mechanisms.

Yet, this definition needs clarification. What is the exact meaning of the phrase “same genetic/developmental mechanisms”? Do we classify two different mutations within the same (causal) gene as convergence or parallelism? As Christin et al. correctly argue, two mutations, even when occurring in the same gene, may determine the same phenotype through entirely different mechanisms (Christin et al., 2010). However, Weng and Noel adopt a definition of parallelism and convergence that is based on the enzymatic activity, stating: “*When ancestral descendants possessing distinct biochemical activities but a shared structural lineage nevertheless contemporarily evolve to synthesize the same metabolite, the term parallel evolution is used. When distinct protein structures sharing no structural similarity result in the synthesis of the same metabolite, the term convergent evolution is employed*” (Weng and Noel, 2013). While this highly interesting paper makes many insightful observations concerning the evolution of metabolism, we feel that, given that it considers only the enzymatic activity and the structural folds, its conclusions may not be scalable to the entirety of evolutionary scenarios. Moreover, the definitions that they provide are rather complicated and seemingly overlapping. We would thus rather urge adoption of more simple terminology. For example, Washburn et al. prefer to use the term “convergence/convergent evolution” in a very general way, stating that its definition is the “*appearance of similar phenotypes in distinct evolutionary lineages […] in a way that cannot be easily explained by descent from a common ancestor*” (Washburn et al., 2016). Todd Barkman uses “convergent evolution” to simply denote the presence of similar phenotypes, independently of the taxonomic position of the species involved or of the underlying genetic mechanisms that lead to it. He states that *“[…] convergent evolution has resulted in the independent origins of many traits dispersed throughout the tree of life. Whereas some convergent traits are known to be generated via similar developmental or biochemical pathways, others arise from different paths […]*” (Huang et al., 2016). Also, in a more recent study, he uses the term “convergence” as an umbrella term to denote a spectrum of different phenomena, stating: *“[…] at one end of the spectrum, convergent traits may arise from different pathways, genes, and sets of mutations, whereas at the other, in principle, it is possible to have the same pathway, generate a similar phenotype in independent lineages that are realized by orthologous genes that acquired their novel functions by identical mutations to the same ancestral nucleotides […]”* (O'Donnell et al., 2021).

Having presented the cases made regarding the dualism between “convergence” and “parallelism”, and the current thinking that emerged from state‐of‐the‐art genetics studies, the idea of having a “spectrum” of convergent phenomena is to us the most valid. James et al have advocated adoption of the term “replicated evolution” as a single, unique term to denote the presence of similar phenotypes in distinct lineages, asking the community to define in any case the genetic and evolutionary causes underlying this similarity (James et al., 2023). While we find this an excellent idea, we firmly believe, as Barkman, that the term convergent evolution is a better catch‐all term and therefore will use this when referring to the general phenomenon of the independent evolution (Table 1) of phenotypic similarity, irrespective of (i) the phylogenetic distance of the organisms involved; (ii) the phenotypic ancestral states; and (iii) the possible homology of the underlying genetic or developmental mechanisms.

## PROCESSES UNDERLYING PHENOTYPIC SIMILARITY

**Figure 2:**
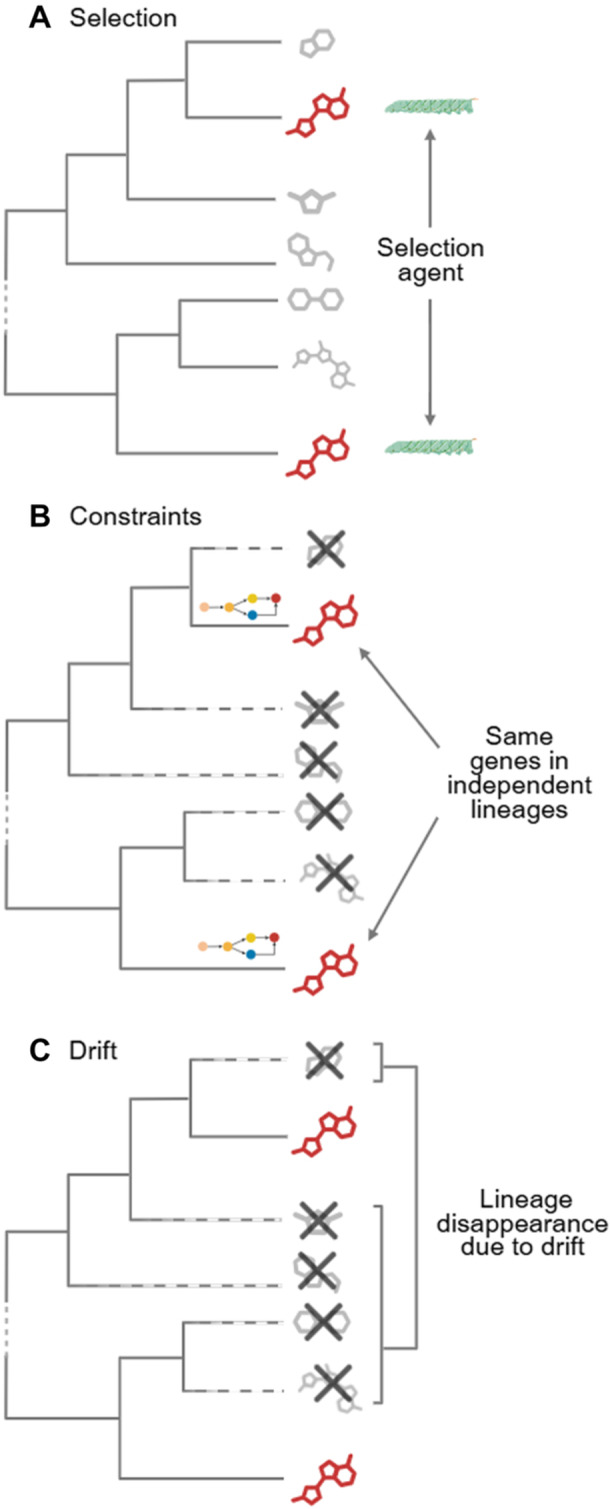
Causes of convergence (*sensu lato*) **(A**–**C)** Homoplasy in plant chemical phenotypes can emerge from a combination of: **(A)** natural selection (represented here by a herbivore vulnerable to the red molecule); **(B)** physical/developmental constraints, which limit the number of evolutionary outcomes to a few lineages, where the similarity is driven, most often, by homologous genes; and **(C)** drift, when the occurrence or disappearance of specific lineages is determined essentially by random events. These three evolutionary events often interact, in different combinations, in generating convergent phenotypes in independent lineages. Figure created using BioRender.

Having established a working definition of convergent evolution, it is important to understand how it can occur. In their excellent review, Washburn et al. propose that there are at least three evolutionary processes that can give rise to similar phenotypes, namely: (i) Similar selective forces driving trait development in multiple lineages; (ii) underlying constraints may force trait evolution; and (iii) a traits‐repeated emergence due to genetic drift (Washburn et al., 2016). These are, of course, by no means mutually exclusive, and most cases of convergence probably result from a mixture of all three (Figure 2). As they themselves also state, common ancestry is of course another common (confounding) cause of convergent evolution. That aside, it is important to discuss all three of these processes, since many studies appear to (almost) exclusively focus on selection (see, for example, (Negin and Jander, 2023)). That said, selection is a good place to start, and the canonical example provided by the C4 trait provides an excellent case. As mentioned above, this trait has independently arisen at least 61 times, with different enzymes, biochemical pathways, and anatomic configurations being used to the same end (Sage et al., 2012). Similar examples are provided by the crystalline lenses of birds and mammals (Schwab et al., 2012), and the independent evolution of nylonases (Prijambada et al., 1995). In all instances, there is clear evidence of more than one viable solution to the problem, with similar outcomes being afforded despite considerable differences in the details (Washburn et al., 2016). There are, however, a number of constraints that reduce the number of potential genetic solutions to a given problem. One strong example of this is the preferential retention of some gene classes over others following whole‐genome duplication (WGD) events. Such a preferential retention represents a clear constraint to the possibility space of selection (Freeling, 2009; James et al., 2023; Beringer et al., 2024). The sporadic presence of caffeine across Angiosperms, for example, can be largely reconducted to the fate—in terms of retention or loss—of specific SABATH gene family members, so that it is basically the genomic constitution (at the time of selection) that constrains the subtype of methyltranferases to be co‐opted for caffeine synthesis (Vignale et al., 2025). However, the genomic space of possibilities is not the only constraint: as James et al. discuss, there are a range of developmental, genetic and physical constraints. For example, mutation limitation can arise from low mutation rates or in instances in which developmental processes impose limitations on the steps needed to build an organism (Charlesworth et al., 1982; Smith et al., 1985). However, the availability of mutations and favorable environments can lead to rapid genetic diversification such as adaptive radiations (Todesco et al., 2020). Physical and genetic constraints can be severe, leading to similarity among unrelated organisms (Losos, 2011; Laruson et al., 2020); however, genome duplication is a powerful mechanism to overcome this (Walsh and Lynch, 2018) and is particularly prominent in plant genomes (Zhang, 2003; Fernie and Tohge, 2017). A striking example of this is provided by the ABC genes of floral development that evolved only once (Bowman et al., 1989). However, in Angiosperms, proliferation of paralogs of the B class led to the independent evolution of modified petals in Ranunculales (Zhang et al., 2013) and orchids (Su et al., 2013; Pan et al., 2014). The preponderance of gene clusters in plant‐specialized metabolism (Tohge et al., 2016; Nutzmann et al., 2018; Zhan et al., 2022), many of which have arisen from gene duplication, followed by neofunctionalization, suggests that this is also an important mechanism underlying the convergent evolution in plant metabolism. Genetic drift can also explain many examples of convergent evolution, with the antifreeze proteins of Arctic and Antarctic fishes probably starting with mutations that were later co‐opted and expanded (Chen et al., 1997; Fletcher et al., 2001). Indeed, gene networks can continue to evolve by drift and selection, such that the effects of drift and stabilizing selection may lead to erratic but nonetheless bounded evolutionary trajectories (James et al., 2023). Interestingly, population size comes into play here, since when populations are small, drift can overcome the deterministic effects of selection, except in the case of strongly selected genes (James et al., 2023).

Having defined the possible mechanisms leading to convergent evolution, we need to confess that it is very difficult to understand which of the above processes is responsible in cases of phenotypic similarity. As we already alluded to selection, constraints and drift are not mutually exclusive and often all contribute, in different proportions, to homoplasy. This is particularly true in studies of metabolic convergence in plants. Therefore, we cannot really state that “paper X demonstrates a convergent metabolic phenotype because of selection”; we can simply claim that, in general, papers reporting convergence of metabolic phenotypes in plants seem to ascribe the phenomenon almost exclusively to selection. Indeed, Negin and Jander seem to suggest that evolution of chemical defenses is all based on selection (i.e., based on the selective pressures represented by the presence of insects and other herbivores (Negin and Jander, 2023)). We, however, think that the story is more complex. In support of this theory, we list several factors that may render a particular metabolite or metabolite class to be particularly prone to convergence across distant lineages, namely:

1. Multifunctionality of the metabolite/metabolite class: If a metabolite has several roles (e.g., roles beyond defense), then (probabilistically) it can be selected multiple times, in multiple lineages, for different reasons (Xu and Gaquerel, 2025). Examples of such compounds are the cyanogenic glycosides and benzoxazinoids (Sanchez‐Perez and Neilson, 2024; Florean et al., 2025);

2. Short/linear pathways, relatively simple chemical structures and multifunctionality should allow a metabolite to emerge sporadically in plant evolution, with caffeine being perhaps a strong example of this case (Huang et al., 2016; O'Donnell et al., 2021; Jia et al., 2026; Vignale et al., 2025);

3. Logically, proximity of the metabolites to primary metabolism, which is generally highly conserved, renders convergent evolution in distant lineages more frequent. For example, the cyanogenic glycosides are only one or two enzymatic steps away from their amino acid primary metabolism precursors (Sanchez‐Perez and Neilson, 2024).

What is debatable, however, is the question as to whether complexity is a strong limit to the development of convergence. C4 and CAM are undeniably complex adaptations (both anatomically and physiologically), but have evolved independently multiple times in plants (Heyduk et al., 2019). That said, what does appear to be a limit to establishment of convergence is chemical complexity. This includes complexity of the metabolite *per se* and of its biosynthetic pathway, in terms of its chemistry, enzymology, and subcellular compartmentalization. Examples for such taxonomically restricted compounds are paclitaxel (Zhang et al., 2023; Fernie et al., 2024; McClune et al., 2025) and vinblastine (Geu‐Flores et al., 2012; Zhang et al., 2022).

## EXAMPLES OF CONVERGENCE *SENSU LATO* IN THE EVOLUTION OF PLANT METABOLISM

Having introduced the terminology, we highlight below recent studies that have elucidated the genetic basis of metabolites' convergence. We present first plant domestication, as an example of a rapid evolutionary process that provides many cases of convergent metabolic phenotypes. Convergence has also been observed in multiple classes of specialized metabolites whose sporadic distribution in the plant kingdom makes the hypothesis of shared ancestry unlikely. The cases presented here are not intended as a comprehensive survey of all instances of metabolic convergent evolution in plants (for a detailed account, see Pichersky and Lewinsohn (2011); Table 2), but rather represent examples in which we attempt to analyze the varying contributions of selection, constraints and drift in determining convergent metabolic outcomes.

**Table 2:** Examples of convergence in metabolic phenotypes across the green lineage

Shared trait(s)	Plant species/families in which phenotypic similarity is observed	MYA separating the taxa*	Mechanism, e.g., (shared ancestry, mutations at orthologous/non‐orthologous loci, HGT, etc.)	References
Acylsugars	Genera *Solanum*, *Datura*, *Nicotiana*, *Salpiglossis* (Solanaceae).	9.3 (divergence *S. nigrum*–*S. pennellii*)	For the acylsucrose hydrolase step, independent recruitment of non‐orthologous genes.	Lou et al. (2021)
Benzoxazinoids	Poaceae (monocots), Acanthaceae, and Lamiaceae (eudicots), but also sporadically present in other families of eudicots (Schullehner et al., 2008).	110 (LCA of Poaceae, (Gallaher et al., 2022), 61 between Acanthaceae and Lamiaceae	Shared ancestry (monophyletic origin within Poaceae), independent recruitment of different genes (different families) for all biosynthetic steps between monocot and eudicot lineages, independent recruitment of almost all genes (e.g., different CYP families) between the eudicot lineages.	(Dutartre et al. 2012; Florean et al. 2023)
Berberine	*Berberis* spp., *Coptis* spp. (Ranunculales) and *Phellodendron* spp. (Sapindales).	142	Recruitment of non‐homologous genes with pathway intermediates largely conserved.	(Xu et al. 2024)
Cannabinoids	*Cannabis sativa* (Cannabaceae) and *Helichrysum umbraculigerum* (Asteraceae).	125	Independent recruitment of non‐homologous genes showing the same enzymatic activities in *C. sativa* and *H. umbraculigerum.*	(Berman et al. 2023)
Cucurbitacins	Cucurbitaceae: *Cucumis sativus* (cucumber), *C. melo* (melon), and *Citrullus lanatus* (watermelon).	20.4	Independent loss‐of‐function mutations in orthologous TF genes regulating the expression of cucurbitacin biosynthetic genes.	(Zhou et al. 2016)
Cyanogenic glycosides	Ferns and seed plants, but present also in arthropods.	405 (Ferns‐Prunus divergence)	Mostly independent recruitment of non‐orthologous genes, with only the first step (conversion of amino acid into aldoxime) catalyzed by a CYP79 gene in all seed plants.	(Beran et al. 2019; Sanchez‐Perez and Neilson 2024)
3‐Deoxyanthocyanidins	Mosses, ferns, and cereals.	488 (Bryophyta‐*Sorghum*); 405 (Ferns‐*Sorghum*)	Independent evolution of non‐homologous loci (canonical late anthocyanin pathway genes not present in mosses and ferns).	(Piatkowski et al. 2020)
Flavones	All major land plant lineages.	60 (Apiaceae divergence)	Recruitment of a non‐homologous flavone synthase gene in Apiaceae.	(Martens and Mithofer 2005)
Iridoid monoterpenes	*Nepeta* spp. (Lamiaceae) and insects.	1,600	Non‐homologous set of biosynthetic genes, but pathway intermediates conserved.	(Lichman et al. 2020; Kollner et al. 2022)
Momilactones	Bryophytes (*Calohypnum plumiforme)* and Poaceae (*Oryza sativa*, wild rice species, and *Echinochloa crus‐galli*).	480	Mosaic origin of homologous and non‐homologous genes located in a conserved biosynthetic gene cluster (BGC).	(Mao et al. 2020)
Nesocodin	*Nesocodon mauritianus* (Campanulaceae) and *Jaltomata herrerae* (Solanaceae).	104	Non‐homologous biosynthetic genes.	(Roy et al. 2022)
Pyrrolizidine alkaloids	Apocynaceae, Asteraceae, Boraginaceae, *Crotalaria* spp.(Fabaceae), some genera of Orchidaceae, and other sporadic occurrences in various Angiosperm families.	46 (Apocynaceae clade age)	Shared ancestry of homospermidine synthase (*HSS*), but unclear evolutionary patterns for the remaining pathway genes.	(Reimann et al. 2004; Smith et al. 2025)
Tropane alkaloids (tropine synthesis step)	Solanaceae and Erythroxylaceae.	118	Independent recruitment of non‐homologous genes.	(Jirschitzka et al. 2012; Tian et al. 2022)
Xanthine alkaloids (caffeine)	*Coffea* spp., *Citrus* spp., *Camellia sinensis* (tea), *Ilex paraguariensis* (yerba mate).	114 (*Coffea*‐*Camellia*), 125 (*Coffea*‐*Citrus*), 102 (*Coffea‐Ilex*)	Recruitment of paralogous genes from the SABATH methyltransferase family, with pathway intermediates not conserved.	(Kato et al. 2000; Huang et al. 2016; O'Donnell et al. 2021; Vignale et al. 2025)

* If not specified otherwise, divergence times have been estimated with TimeTree 5 (timetree.org, Kumar et al. 2022).

### Domestication

Some of the best‐studied examples of convergent traits come from studies of plant domestication. During the process of genetic adaptation to human needs, wild species underwent a series of morphological and physiological changes to become apt for cultivation and consumption. The loss of seed dormancy, for example, is an important transition accompanying the domestication of many seed crops (dormancy is beneficial in the wild, but uniform and fast germination is required in cultivated plants). In soybean, the study of the stay green trait revealed that the underlying locus (*G*) also controlled seed dormancy. The causal gene, encoding a protease that affects abscisic acid (ABA) levels ultimately, was located in a selective sweep region, not only in soybean but also in rice, tomato, and Arabidopsis, with its role in determining seed dormancy loss conserved across these species. In other words, the orthologs of G were subjected to parallel selection during domestication in different families (Fabaceae, Poaceae, and Solanaceae) (Wang et al., 2018). Other traditional examples of phenotypic similarity were related to some of the typical traits of domestication syndrome (e.g., seed shattering in sorghum, maize, and rice (Paterson et al., 1995; Lin et al., 2012)). In sorghum and maize, loss of shattering was achieved through knockdown mutations in *Shattering1* (*Sh1*). The same gene played an accessory role in determining the non‐shattering phenotype also in rice, in addition to other major genes (Konishi et al., 2006; Li et al., 2006; Zhou et al., 2012; Yoon et al., 2014), while in wheat, the phenotype is controlled by the *Q* gene, non‐orthologous to *Sh1* (Lin et al., 2012; Gaut, 2015). The finding suggests that, at least for sorghum and maize, the genetic space of possible genetic solutions leading to the non‐shattering phenotype was constrained through mutations in *Sh1* (although the contribution of genetic drift cannot be excluded; see the discussion in Gaut (2015)). Some of the early results of a shared genetic origin in several domestication phenotypes in the grasses (Paterson et al., 1995) have been re‐assessed more recently, with studies at higher genetic resolution revealing several cases of phenotypic similarity driven by non‐homologous loci (Sang, 2009; Sood et al., 2009; Di Vittori et al., 2019; Woodhouse and Hufford, 2019; Yu and Kellogg, 2024). Beyond structural traits, domestication also targeted metabolic pathways. Examples include fruit color, fragrance, and the dwarfing habit of cereals, with the latter largely achieved through mutations in the gibberellin biosynthetic pathway (see below). Culinary preferences also drove selection in case of the acquisition of the sticky texture (glutinous) that some cereal seeds (and pseudocereals like amaranth) assume when cooked. This sticky texture is due to a reduced content of amylose, a component of starch. In all species analyzed so far (rice, maize, sorghum, wheat, barley, millet, and amaranth), the “glutinous” trait was acquired through different changes at the orthologous *waxy* locus, encoding the granule‐bound starch synthase I (GBSSI), the enzyme responsible for the synthesis of amylose (Gaur et al., 2024).

As mentioned above, the dwarfing trait in cereal crops can be essentially reconducted to various perturbations of gibberellin metabolism and provides another typical example of the domestication syndrome (Peng et al., 1999; Hedden, 2003). Many rice (Spielmeyer et al., 2002; Asano et al., 2011) and barley (Jia et al., 2009; Xu et al., 2017) dwarf and semi‐dwarf varieties, for example, have achieved shorter stems through loss‐of‐function mutations in the orthologous gene *gibberellin 20 oxidase 2 (GA20ox‐2)*, abolishing synthesis of gibberellins (Xie et al., 2024). In wheat, on the other hand, most of the selected dwarfing mutations map to the *Reduced height‐1 (Rht‐B1 and Rht‐D1)* loci, which encode truncated DELLA proteins that reduce sensitivity to gibberellins (Peng et al., 1999). There are clearly many more metabolism‐related genes that have been targeted, directly or indirectly, by domestication (for a survey, see Alseekh et al. (2021)), and, after decades of intense discussions, there are few doubts today that phenotypic convergence during domestication can be reached either through mutations in different genes or from recruitment of homologous genes (for a summary of the debate, see Gaut (2015); Pickersgill, (2018)).

A central question is why certain genes seem to be particularly prone to be *reused*—more than others—in different evolutionary lineages to give rise to convergent phenotypes. In other words, is it possible to identify inherent genetic characteristics that promoted the shared adoption of some particular (metabolic) genes resulting in convergent phenotypes? There are no simple answers to this question, and much, of course, depends on the phylogenetic scale at which the problem is analyzed. Intuitively, changes in orthologous loci giving rise to convergent phenotypes are more frequent within the same species or in closely related species (within the same family), to then become rare occurrences at higher phylogenetic distances simply because molecular convergence requires some degree of genomic similarity, and orthologs might be simply not present when comparing different species above the family level. Thus, although there are no absolute criteria for predicting the probability of gene reuse, except for phylogenetic distance, at least four predisposing conditions have emerged from empirical studies, and we briefly describe them below.

#### Target size

Target size is defined as the number of genes capable of producing a given phenotype (Gompel and Prud'homme, 2009). For example, enzymatic genes in linear/simple pathways have an increased possibility of becoming fixed in different species/populations because they often exert strong, direct control on the associated biochemical trait, that is, the accumulation of the downstream metabolite. This is likely the case for the repeated evolution of the *waxy* phenotype in cereals, a trait governed by a simple genetic architecture in all species analyzed so far (Nakamura et al., 1995; Patron et al., 2002; Fan et al., 2009; Hunt et al., 2010; Misra et al., 2018). The evolution of the glutinous texture thus followed an almost obligate path in multiple species, simply because the *waxy* alleles had a large effect on the manifestation of the trait, and their probability of being fixed should have been considerably higher with respect to mutations with a weaker effect on the phenotype.

#### Network position

“Input–Output” genes, such as transcription factors at the nodal position in regulatory networks, integrate multiple environmental or developmental signals through upstream signaling pathways and regulate the expression of several downstream genes, which act synergistically in the expression of a specific phenotype. This explains the repeated selection of a few, out of many, “preferred” photoperiod and flowering time genes during domestication in multiple species, for example, *VRN2*, *FLOWERING LOCUS C,* and *FLOWERING LOCUS T* (Gaudinier and Blackman, 2020).

#### Pleiotropy

Alleles with minimal pleiotropic effects are more likely to become fixed in multiple lineages. The full spectrum of phenotypes generated by a gene with large pleiotropic effects is rarely all beneficial. Hence, the homologous genes that we observe today, which underlie convergent phenotypes, might simply be the outcomes of a preferential over‐representation of alleles with low pleiotropic effects. This might be the case for the changes in flower pigment intensity observed during domestication, which have been mostly achieved through fixation in the *cis*‐regulatory (non‐coding) regions of MYB TF genes (Wu et al., 2022; Marin‐Recinos and Pucker, 2024). Two arguments have been provided in support of this gene reuse during the flower transitions in plant domestication. The first is that mutations in the promoter and in other non‐coding regions are rarely deleterious, as they usually affect the timing or tissue‐specific expression. As such, they are unlikely to induce pervasive pleiotropic effects affecting distant plant parts (Streisfeld and Rausher, 2011); the second is that MYB TFs, in comparison to other TF families, traditionally also involved in coloration phenotypes (e.g., bHLH, (Albert et al., 2021) and WD40 (Parker et al., 2024)), have a lower degree of pleiotropy, given that MYBs, when mutated, usually have tissue/organ‐specific effects (Butelli et al., 2012; Chopy et al., 2023).

#### Standing variation

Selection can act on pre‐existing alleles present at low frequency in wild populations of the different species/lineages involved (without exerting strong deleterious effects). In response to some intervening selection pressures, these low‐frequency alleles can become beneficial and increase in frequency in all species involved and may ultimately become fixed. In these conditions, the fixation of alleles that are already present in the wild gene pool is clearly favored with respect to the fixation of newly emerging beneficial mutations. While adaptation from standing variation is well documented in plant domestication (e.g., *teosinte branched* in maize (Studer et al., 2011)), evidence for ortholog reuse in plant metabolism via this mechanism remains rare, and more research is thus needed in this area.

### Momilactone biosynthesis

Momilactones are labdane‐derived diterpenoids that function both as allelochemicals and as defensive compounds against microbial pathogens. They were originally discovered in Asian rice (*Oryza sativa*, Kato et al. (1973)), in wild rice species (*O. barthii*, *O. glumaepatula*, *O. meridionalis*, and *O. rufipogon* (Miyamoto et al., 2016)) but were later also isolated in *Echinochloa crus‐galli* (barnyard grass) and from the bryophyte *Calohypnum plumiforme*, a moss whose lineage diverged from the rest of other Embryophytes around 440–460 MYA (Nozaki et al., 2007; Mao et al., 2020). The early investigations on the genes controlling the accumulation of momilactones in rice led to the discovery of one of the first biosynthetic clusters in plants (Wilderman et al., 2004; Shimura et al., 2007). Remarkably, a cluster, containing genes with the same enzymatic activities, was later also identified in the evolutionarily distant moss *C. plumiforme* (Mao et al., 2020). What is interesting here, in terms of understanding the establishment of metabolic convergence between such distant species, is that some of the genes in the cluster are clearly non‐homologous (e.g., those encoding the cytochrome P450 monooxygenases and the terpene cyclases), while one, the short‐chain dehydrogenase catalyzing the final step (the momilactone synthase, MAS), is > 50% similar between rice and *C. plumiforme*, and was shown to catalyze the same reaction in the two species (Mao et al., 2020). Thus, the pathway underlying the convergent appearance of momilactone biosynthesis is a mixed mosaic of homologous and non‐homologous biosynthetic genes. Convergence is evident not only at the gene level but also at the level of genomic organization. In both the moss and rice, all biosynthetic genes are clustered within contiguous genomic regions: four genes in *C. plumiforme* and five to six, depending on the species, in the grasses, spanning from 57 kb in *E. crus‐galli* to around 150–180 kb in rice and *C. plumiforme*. The evolutionary drivers for the accumulation of momilactones and the formation of the biosynthetic cluster in such distant lineages remain unknown, but a similarity in the ecological niches of the two species, implying similar selective pressures, likely contributed to it. Additional selective advantages might have included coinheritance and coexpression of the clustered biosynthetic genes, facilitating efficient pathway regulation. Conversely, a dispersed genomic organization of the biosynthetic genes may have been disfavored, as it could have led to the accumulation of potentially toxic intermediates. Indeed, toxicity for some of the early momilactone precursors was demonstrated in rice (Xu et al., 2012). Taken together, momilactone biosynthesis exemplifies metabolic convergence generated by the recruitment of both homologous and non‐homologous biosynthetic genes. The persistence of these clusters, maintained across more than 400 MYA of evolution, is probably explained by a complex interplay of positive and purifying (negative) selection.

### Iridoid biosynthesis

Perhaps one of the most striking cases of convergence is the occurrence of the same set of specialized metabolites in plants and insects. Since the initial observations of this phenomenon, made approximately 50 years ago with the co‐occurrence of benzoquinones and anthraquinones in arthropods and higher plants (Rodriguez and Levin, 1976), many other cases have been reported (Beran et al., 2019). Among these, iridoid monoterpenes represent one of the best‐studied examples. Iridoids are produced in several members of the Lamiaceae (the mint family, Boachon et al. (2018)) and in several orders of insects (Coleoptera, Hymnoptera, and Hemiptera; Beran et al. (2019)). In plants, they function primarily as repellents against herbivores, while in insects, they both serve as repellents and as sex pheromones (Kollner et al., 2022). The co‐occurrence of identical metabolites in such distant taxa makes the recruitment of homologous genes highly unlikely, and, in fact, the biosynthetic genes in the two species (the plant *Nepeta cataria* and the pea aphid, *Acyrthosiphon pisum*) are totally unrelated. What makes this case particularly remarkable is that, despite the lack of homology between the biosynthetic genes, the pathway to nepelactone, the final product in the pathway, proceeds exactly through the same set of intermediates in both species (Lichman et al., 2020; Kollner et al., 2022). How was it possible that the same sequence of metabolic intermediates evolved in organisms so evolutionarily distant? Iridoids have strong repellent activities toward ants, beetles, and other phytophagous insects and, as such, their presence is beneficial for both plants and insects to defend against their respective predators (Eisner, 1964). In addition to their defensive role, in aphids, the iridoids nepelactol and nepetalactone are synthesized and released as volatiles by adult females to act as sex pheromones (male attractants (Kollner et al., 2022)). It thus seems likely that the convergence at the pathway level, despite the different enzymology, underscores the strength of selective pressures favoring iridoid production. In this process of distant evolutionary convergence, genetic or developmental constraints were probably negligible, given that the genes and the enzymology of the pathways are different. Instead, iridoid biosynthesis exemplifies how similar ecological challenges can drive the independent evolution of identical metabolites across deeply divergent lineages (Figure 2).

### Floral fragrance

The flower of Angiosperms has been, historically, one of the main focus of plant evolutionary biology in studies of convergence. More than any other organ, the flower displays an impressive variation in morphological, developmental, and biochemical traits. Consequently, both comparative phylogenetics and evo–devo approaches have been used to determine whether similar floral traits in distinct lineages represent true convergence, and thus ascribed to homoplasy, or are derived instead from shared ancestry (Papadopulos et al., 2013; Ng and Smith, 2016; Smith and Kriebel, 2018; Simon‐Porcar et al., 2024). In the field of plant metabolism, the chemical composition of floral scent was among the first traits analyzed in terms of convergence, as the emission of volatiles is clearly shaped by pollinator‐mediated selection. Early studies of neotropical species from different families (Bignoniaceae, Cactaceae, and Cleomaceae), all pollinated by bats, had a highly similar volatile composition of their floral scents, characterized by sulfide‐containing compounds (Knudsen and Tollsten, 1995). Although these initial studies lacked detailed genetic characterization of the loci underlying the phenotypic convergence, more recent work in *Capsella* has uncovered the genetic basis of shifts in floral fragrance (Wozniak et al., 2024). In *Capsella*, the ancestral outcrossing populations independently gave rise to two self‐fertile lineages: the first transition gave rise to the selfer *C. orientalis* (Bachmann et al., 2019), while the second, more recent transition to self‐fertilization (dated to 20–50,000 years ago) gave rise to *C. rubella* (Guo et al., 2009). The floral volatiles of these two selfer lineages are highly similar to each other, but differ drastically from the modern, outbreeder descendant (*C. grandiflora*) of the ancestral self‐incompatible population. A key difference between the two lineages is the drastic reduction of β‐ocimene in the selfers. This convergent change was attributed to a region on chromosome 7 in the case of *C. rubella* and on chromosome 4 in the case of *C. orientalis*. The case of *Capsella* is a great example that phenotypic similarity, even within a single genus, may not be due, as it is usually expected, to shared ancestry. Instead, independent transitions to self‐fertilization were accompanied by convergent shifts in floral volatiles, driven by mutations at different loci (Wozniak et al., 2024).

### Tropane alkaloids

Chemotaxonomic studies have long reported the presence of tropane alkaloids (TAs) across disparate families of land plants (Robinson, 1968). These nitrogen‐containing heterocycles are widely distributed in Angiosperms and have been reported so far in several species of Erythroxylaceae, Convolvulaceae, Brassicaceae, Proteaceae, and Euphorbiaceae (Griffin and Lin, 2000). Their biosynthesis has been extensively studied in Solanaceae, particularly in the genera *Atropa*, *Brugmansia*, *Datura*, *Duboisia,* and *Hyoscyamus* (Perez‐Mesa and Roda, 2025), and in Erythroxylaceae, the family that includes cocaine‐producing plants. Early comparative studies between *Erythroxylum coca* and TA‐accumulating Solanaceae species already pointed to the independent recruitment of non‐homologous genes for a key step in the pathway: the reduction of the cyclic ketone (ecgonone/tropinone) to its corresponding alcohol (ecgonine/tropine) (Jirschitzka et al., 2012). More recent phylogenetic and genomic studies have extended this observation, showing that basically all enzymes in TA biosynthesis, from the initial conversion of arginine into putrescine, to the synthesis of ecgonone (the last common precursor before the pathway's divergence toward cocaine in Erythroxylaceae and hyoscyamine/scopolamine in Solanaceae) arose through independent gene recruitment (Chavez et al., 2022; Wang et al., 2023). This pattern was also present for those steps catalyzed by enzymes of the same supergene family. For example, the conversion of malonyl‐CoA into 3‐oxoglutaric acid is catalyzed by polyketide synthases (PKSs) in both *Erythroxylum novogranatense* and *Anisodus acutangulus* (Solanaceae). Yet, the PKSs involved belong to distinct clades: the neo‐pyrrolidine ketide synthases (Neo‐PYKS clade) in Malpighiales (including Erythroxylaceae) and the canonical pyrrolidine ketide synthases (PYKS clade) in Solanales. Structural analyses of the two neo‐PYKSs characterized in *E. novogranatense* (*En*PKS1/2) showed a different active site architecture with respect to the *A. acutangulus* PYKS (*AaPYKS1*) involved in hyoscyamine biosynthesis, although the two enzymes catalyze the same identical reaction in Erythroxylaceae and Solanaceae (i.e., the synthesis of 3‐oxoglutaric acid from two units of malonyl‐CoA). This was an exemplary case of catalytic convergence of PKSs from two distant plant families, achieved through relatively minor amino acid changes altering the size of the catalytic pocket (a similar case of convergence, where different residues allow the correct positioning of the same substrate in the active site, has also been reported in the xanthine methyltransferase DXMT from *Coffea canephora* and in the caffeine synthase CS3 from *Ilex paraguariensis* (Vignale et al., 2025)). The different phylogenetic distribution of the Erythroxylaceae PKS1/2 from the Solanaceae*‐*specific *PYKS* and the non‐interchangeable nature of the amino acid replacements in the active site make the hypothesis of independent recruitment from different ancestral non‐orthologous PKS genes better supported with respect to the alternative hypothesis of a shared ancestry from a single precursor gene (Huang et al., 2019; Tian et al., 2022). The independent origin of the TA genes is also coherent with the differences in pathway localization between the two families. In Solanaceae, TAs accumulate in the roots and are subsequently transported to the aerial tissues. Within root cells, their biosynthesis also traverses multiple compartments (ER, cytosol, vacuole, and back to cytosol (Srinivasan and Smolke, 2020, 2021)). In contrast, in Erythroxylaceae, active synthesis only occurs in the aerial parts of the plant (Jirschitzka et al., 2012). Thus, the convergence of TA biosynthesis in such disparate lineages probably reflects selective pressure not only on enzyme function but also on tissue specificity, subcellular localization, and expression patterns during plant development. These distinct requirements likely favored the recruitment of entirely different gene sets, resulting in convergent metabolic outcomes despite independent genetic origins (Chavez et al., 2024).

### Amanitin biosynthesis

The cyclic octapeptide α‐amanitin is a potent toxin found in several phylogenetically distant genera of poisonous fungi of the order Agaricales. In addition to the notorious *Amanita phalloides* and the other, equally dangerous, species of the same genus (e.g., *A. rimosa*, *A. virosa,* and many others), species belonging to the genera *Lepiota* and *Galerina*, which diverged from *Amanita* 84 and 139 MYA, respectively, are also known to accumulate α‐amanitin (Walton, 2018). Unlike other cyclic fungal peptides that are synthesized by nonribosomal peptide synthetases (NRPSs, (Gao et al., 2012)), amanitin and the other related amatoxins are synthesized on ribosomes from transcripts encoded by genes of the MSDIN family (Hallen et al., 2007). The propeptide is then posttranslationally processed by a prolyloligopeptidase (POPB), flavin mono‐oxygenase (FMO), and cytochrome P450s (CYP450s) (Luo et al., 2014, 2022). Comparative genomic analysis of representatives from these three genera has reconducted the convergent presence of α‐amanitin to an event of horizontal gene transfer (HGT), which probably occurred in the common ancestor of the three genera. Subsequent divergence was accompanied by gene duplications and genomic rearrangements, resulting in a massive expansion of the pathway in *Amanita*. This expansion underlies the broader diversity of amanitin analogues in *Amanita* compared to *Lepiota* and *Galerina* (Luo et al., 2022). Although the occurrence of HGT was once considered controversial in multicellular organisms (Martin, 2017), and is generally less frequent in eukaryotes than in prokaryotes (McInerney, 2017), well‐supported HGT events have been increasingly reported in animals (Moran and Jarvik, 2010), plants (Ma et al., 2022), and fungi (Slot and Rokas, 2011). HGT is now considered an important mechanism contributing to establishing convergence (McInerney, 2025). A striking example is the evolution of C4 photosynthesis, a convergent adaptation to hot and arid environments in phylogenetically distant plants (Heyduk et al., 2019). Several genes of the C4 pathway, including phosphoenolpyruvate carboxylase (*ppc)* and phosphoenolpyruvatecarboxykinase (*pck*), were horizontally transferred to the grass lineage *Alloteropsis* from other C4 Poaceae that had diverged at least 20 MYA earlier (Christin et al., 2012). More recent studies have widened the impact of grass‐to‐grass gene transfers in *Alloteropsis*, reporting several loci involved in resistance to biotic and abiotic stresses that also originated via HGT (Dunning et al., 2019). Another traditional area for studying the impact of HGT is that of parasitic plants. Parasitism is relatively common in Angiosperms, with more than 4,000 species being reported as obligate or facultative parasites (Westwood et al., 2010). The discovery of horizontally acquired genes in parasitic plants is not surprising, given the close physical contact between the host and the parasite, facilitating DNA transfer (Yang et al., 2016, 2019; Kado and Innan, 2018). Thus, while plant‐to‐plant HGT is now an established phenomenon, particularly in parasitic lineages, but also across the broader group of land plants (Ma et al., 2022), it remains an open question whether convergent metabolic phenotypes in plants can be attributed to episodes of HGTs, in a way similar to the evolution of α‐amanitin biosynthesis in fungi.

## OBSERVING CONVERGENCE IN REAL‐TIME?

Cases of phenotypic convergence can be considered to be closely connected to Stephen Jay Gould's scenario of “replays of the tape of life”. In his book “Wonderful life: the Burgess shale and the nature of history”, Gould hypothesized that “*[…] if we were to replay the tape of life a million times […], I doubt that anything like Homo sapiens would ever evolve again*” (Gould, 1989). Gould was an advocate for the strong role of historical contingency, so that, replays of evolution, even when starting from identical conditions, would have always produced different outcomes. Gould intended “contingency” both as the occurrence of unpredictable events, which can increase, or reduce, the number of possible evolutionary paths, but also as the probability that a precise path would have been taken only if *contingent* (i.e., causally dependent) on some previous historical events. In essence, Gould's view on the stochastic nature of evolutionary events (mutation, drift, and recombination), combined with their contingent effects on the preclusion of specific successive evolutionary paths, would have made the repetition of determined outcomes impossible (Blount et al., 2018). We now know that evolution repeats itself, and the cases of metabolic convergence in plant metabolism presented in this review are just a handful of examples of a multitude of similar traits observed at the molecular, morphological, and behavioral level in all kingdoms of life (McGhee, 2011). Plant (and animal) domestication can be intended as natural field experiments and is perhaps the closest scenario to what Gould had actually in mind when he thought about the “replays of the tape of life”. As we mentioned above, plant domestication offers many examples of convergence, but today, evolutionary biologists have the chance to replay the tape also under more controlled conditions. Comparative approaches and phylogenetics have provided important insights into the developmental basis of similar (homoplasious) phenotypes (Glover et al., 2015; Mahler et al., 2017; Barkman, 2024), and resurrection of ancestral proteins, although still underused in plants, has afforded important insights into how disparate enzymes have acquired, for example, the biochemical capacities to converge on the synthesis of caffeine (Huang et al., 2016; O'Donnell et al., 2021).

An alternative to these approaches, and a way to observe adaptation in “real time” (and thus also possibly convergence), is to let a population of organisms experience a set of conditions imposed and strictly controlled by the researcher (e.g., a nutrient stress, high or low temperatures, etc.), and then follow the evolutionary changes through various generations of selection. This technique, experimental evolution, could allow, for example, to observe if and how convergent phenotypes emerge through generations (Tenaillon et al., 2012). Experimental evolution has been traditionally applied in microbes (Lenski et al., 1991; Dhar et al., 2011) and *Drosophila* (Burke et al., 2010), and when combined with Pool‐Seq, in the so‐called Evolve & Resequence (E&R) studies, has allowed to identify the alleles under selection by comparing their frequencies between the starting and the evolved population (Schlotterer et al., 2015). In plants, experimental evolution studies are rare, but the few conducted so far have provided important results on the nature of adaptation in response to specific ecological factors (Schiestl, 2024). Experimental evolution in plants necessarily faces the problem of longer generation times with respect to bacteria, yeast, or *Drosophila*. Arabidopsis completes its cycle in 6–8 weeks, and the fast‐cycling Brassicas need around 40 d (Williams and Hill, 1986; Iniguez‐Luy et al., 2009). Under these conditions, observation of adaptations from *de novo* mutations requires a genetically homogeneous starting population, and given the average germinal plant mutation rate, estimated between 10E−8 and 10E−11 (Quiroz et al., 2023), it would be necessary to use an extremely large number of individual plants over many generations. Hence, the few experimental evolution studies in plants start from polymorphic populations and are focused on identifying adaptive alleles from the standing variation already present at the beginning of the experiment. Such studies have identified evolutionary patterns in defensive traits, flowering time, and floral fragrance in response to herbivores and pollinators, but were typically limited to few generations (Agrawal et al., 2012; Zust et al., 2012; Gervasi and Schiestl, 2017; Ramos and Schiestl, 2019, 2020). Long‐term agricultural experiments do exist, however, (Moose et al., 2004; Silvertown et al., 2006; Poulton et al., 2024), although they were not initially designed as strictly selection experiments or have not been (yet) analyzed in the frame of an E&R study. The barley composite cross II (CCII) is perhaps the first long‐term agricultural competition experiment that has been the subject of a 60‐generation‐long evolutionary analysis (Landis et al., 2024). In 1929, Harlan and Martini generated the population by crossing, in all combinations, 28 varieties representative of the barley widest phenotypic diversity. The progeny of all these half‐diallel crosses, mixed in equal proportions, was then used as the founding population (Harlan and Martini, 1929). Since then, the harvested seeds were re‐sown to propagate the population for more than 58 generations, with no artificial selection to let the genotypes compete without human intervention. Pool‐Seq analyses showed that, already after a few generations, the population heterozygosity decreased, in parallel with the gradual emergence of a dominant lineage derived from the ancestral North African parents, which were highly adapted to the local conditions of Davis (California, US). The CCII experiment shows that natural selection may act very rapidly, leading a highly polymorphic population to substantial genetic homogenization in around 50 generations (Landis et al., 2024). This is considerably faster than what we previously thought from the archeobotanical and population genomic studies of cereal domestication (Purugganan, 2019). What is important to stress here is that multi‐generational field competition experiments, like the barley CCII, can be considered to be similar to the parallel replays in experimental evolution, where replicated genotypes or populations evolve under identical conditions. Theoretically, if these experiments are repeated in different environments, they could represent true “historical difference experiments” (see (Blount et al., 2018) for a detailed description of these experimental designs). These experiments are ideal scenarios for evaluating the role of different environments, and the related evolutionary histories, in the possible emergence of convergent phenotypes. It may become possible to observe, for example, whether convergent adaptations, also at the metabolic level, arise as a matter of similarity of environmental conditions (Bakhtiari et al., 2021), or, on the contrary, whether populations evolve different solutions to the same stress. Also, historical seed samples (e.g., those from the 5th, 10th, 30th generation, etc.) can be later used as additional replays, to re‐found the population and let it propagate in different environments, to check the repeatability of the final outcomes observed in the initial replay experiment. These approaches have yet to be tested in plants, which are inherently more complex than the usual organisms used in experimental evolution: The selective pressures shaping their evolution are drastically different and depend on the complexity and diversity of their mating systems, ecological niches, and autotrophic lifestyles. However, evolutionary experiments, when conducted in natural environments over a significant number of generations, offer the possibility to observe how natural selection may lead to convergence and understand the basis of the phenotypic similarities. As several studies have shown (Frachon et al., 2017; Mitchell and Whitney, 2018; Desbiez‐Piat et al., 2021; Landis et al., 2024), selection, also for metabolic traits (Zust et al., 2012; Medina‐van Berkum et al., 2025), may be a relatively rapid phenomenon, at least for some traits controlled by strong effect loci, and can thus be observable in ecological time. Also, we have stressed earlier in this review how difficult it can be to determine the contributions of drift and selection in studies of convergence. Multi‐generational studies also have the advantage of distinguishing these two phenomena. If population size and starting genetic diversity are known, in fact, it is possible to model the contribution of drift alone from the observed allele frequency changes, given that frequency shifts due to selection, contrary to those due essentially to drift, are usually correlated over multiple generations (Buffalo and Coop, 2019; Brown and Koenig, 2022).

Clearly, given the complexity and the resources necessary for these evolutionary multi‐location and multi‐generational field trials, the coordination of such large‐scale natural experiments should better implement a decentralized approach involving a large number of farmers (Santamarina et al., 2025). Participatory strategies in agroecological genomic initiatives have also assured access and use of large collection of plant genetic resources in more environments than it would be normally possible in single breeding stations (Bellucci et al., 2021).

## CONCLUDING REMARKS

The recurring emergence of similar metabolic solutions across disparate plant lineages is more than a catalogue of case studies. It is a lens on how evolution navigates chemical space. Convergence in metabolism reveals a constrained but creative search process, in which natural selection, constraint, chance, and other phenomena, like that of HGT (whose importance was long neglected in homoplasy), interact to produce repeatable outcomes. Whether the path runs through shared ancestry, the reuse of nodal regulatory genes, or novel enzymatic innovations, the end point often aligns on a limited set of biochemically tractable strategies. This insight connects evolutionary theory with actionable practice.

Three implications follow. First, convergence provides predictive power. If unrelated taxa repeatedly arrive at the same metabolites, those solutions are likely robust, accessible, and beneficial under defined ecological regimes. Second, convergence highlights tractable entry points for engineering. Short, linear pathways, proximity to primary metabolism, and low pleiotropy identify targets where modification is both feasible and durable. Third, convergence reframes domestication and *de novo* trait design as guided exploration rather than blind search. Standing variation, network position, and pathway architecture can be leveraged to accelerate trait discovery.

As integrative metabolomics, comparative genomics, and ancestral reconstruction mature, the field can move from describing convergent outcomes to testing their causes in historical field evolutionary experiments and forecasting their recurrence. This will deepen our understanding of the rules that shape metabolic diversity and open routes to resilient crops, sustainable chemistries, and informed conservation. Recognition of convergence as a central organizing principle positions plant biology to bridge mechanism, prediction, and application.

## CONFLICTS OF INTEREST

The authors declare no conflicts of interest.

## AUTHOR CONTRIBUTIONS

F.S. and A.R.F. wrote the manuscript. All authors revised the manuscript, and have read and approved the contents of this paper.
